# Quantifying the heritability of glioma using genome-wide complex trait analysis

**DOI:** 10.1038/srep17267

**Published:** 2015-12-02

**Authors:** Ben Kinnersley, Jonathan S. Mitchell, Konstantinos Gousias, Johannes Schramm, Ahmed Idbaih, Marianne Labussière, Yannick Marie, Amithys Rahimian, H.-Erich Wichmann, Stefan Schreiber, Khe Hoang-Xuan, Jean-Yves Delattre, Markus M. Nöthen, Karima Mokhtari, Mark Lathrop, Melissa Bondy, Matthias Simon, Marc Sanson, Richard S. Houlston

**Affiliations:** 1 Division of Genetics and Epidemiology, The Institute of Cancer Research, 15 Cotswold Road, Sutton, Surrey SM2 5NG, UK; 2 Department of Neurosurgery, University of Bonn Medical Center, Sigmund-Freud-Str. 25, 53105 Bonn, Germany; 3 Sorbonne Universités UPMC Univ Paris 06, INSERM CNRS, U1127, UMR 7225, ICM, F-75013 Paris, France; 4 AP-HP, GH Pitié-Salpêtrière, Service de Neurologie Mazarin, 47 bld de l’Hôpital, 75651 Paris, France; 5 Onconeurotek, F-75013 Paris, France; 6 Institute of Epidemiology I, Helmholtz Zentrum München, German Research Center for Environmental Health, Ingolstädter Landstr. 1, 85764 Neuherberg, Germany; 7 Institute of Medical Informatics, Biometry and Epidemiology, Chair of Epidemiology, Ludwig-Maximilians-Universität, Geschwister-Scholl-Platz 1, 80539 Munich, Germany; 8 Institute of Medical Statistics and Epidemiology, Technical University Munich, Germany; 9 1st Medical Department, University Clinic Schleswig-Holstein, Campus Kiel, House 6, Arnold-Heller-Str. 3, 24105 Kiel, Germany; 10 Institute of Clinical Molecular Biology, Christian-Albrechts-University Kiel, Arnold-Heller-Straβe 3, 24105 Kiel, Germany; 11 Institute of Human Genetics, University of Bonn, Bonn, Germany; 12 AP-HP, GH Pitié-Salpêtrière, Laboratoire de neuropathologie R Escourolle, F-75013 Paris, France; 13 Fondation Jean Dausset-CEPH, 27 Rue Juliette Dodu, 75010 Paris, France; 14 Génome Québec, Department of Human Genetics, McGill University, Montreal, Quebec, H3A 0G1, Canada; 15 Department of Pediatrics, Division of Hematology-Oncology, Dan L. Duncan Cancer Center, Baylor College of Medicine, Houston, Texas, USA

## Abstract

Genome-wide association studies (GWAS) have successfully identified a number of common single-nucleotide polymorphisms (SNPs) influencing glioma risk. While these SNPs only explain a small proportion of the genetic risk it is unclear how much is left to be detected by other, yet to be identified, common SNPs. Therefore, we applied Genome-Wide Complex Trait Analysis (GCTA) to three GWAS datasets totalling 3,373 cases and 4,571 controls and performed a meta-analysis to estimate the heritability of glioma. Our results identify heritability estimates of 25% (95% CI: 20–31%, *P* = 1.15 × 10^−17^) for all forms of glioma - 26% (95% CI: 17–35%, *P* = 1.05 × 10^−8^) for glioblastoma multiforme (GBM) and 25% (95% CI: 17–32%, *P* = 1.26 × 10^−10^) for non-GBM tumors. This is a substantial increase from the genetic variance identified by the currently identified GWAS risk loci (~6% of common heritability), indicating that most of the heritable risk attributable to common genetic variants remains to be identified.

Gliomas account for ~40% of all primary brain tumours and are responsible for around 13,000 cancer-related deaths in the USA each year^1^. Gliomas are defined in part by malignancy grade (*e.g*. pilocytic astrocytoma WHO grade I, diffuse ‘low grade’ glioma WHO grade II, anaplastic glioma WHO grade III and glioblastoma (GBM) WHO grade IV can be distinguished^2^). Most gliomas carry a poor prognosis irrespective of treatment, with the most common form of glioma, glioblastoma (GBM), having a median overall survival of ~15 months^3^,^4^.

While the glioma subtypes have distinct molecular profiles resulting from different etiological pathways^5^, no environmental factor has been consistently linked to disease risk except exposure to ionizing radiation, which accounts for few cases^1^. Evidence for inherited genetic susceptibility to glioma is provided by a number of rare inherited cancer syndromes, including Turcot’s and Li–Fraumeni syndromes, and neurofibromatosis^6^. Even collectively these syndromes however account for little of the two-fold increased risk of glioma seen in relatives of glioma patients^1^,^7^.

Recent genome-wide association studies (GWAS) have provided unambiguous evidence for common genetic susceptibility to glioma - identified single nucleotide polymorphisms (SNPs) at 5p15.33 (*TERT*), 7p11.2 (*EGFR*) 8q24.21 (*CCDC26*), 9p21.3 (*CDKN2A/CDKN2B*), 11q23.3 (*PHLDB1*) and 20q13.33 (*RTEL1*) associated with risk^8^,^9^,^10^. It is not clear however how much of the heritability of glioma ascribable to common variation these risk SNPs explain.

Testing SNPs individually for an association in GWAS necessitates the imposition of a very stringent *P*-value to address the issue of multiple testing (*i.e.* conventionally *P* ≤ 5.0 × 10^−8^). While this reduces false positives it can result in true associations being missed. Genome-wide Complex Trait Analysis (GCTA) allows the contribution of all SNPs in a GWAS to be simultaneously evaluated in estimating heritability^11^,^12^,^13^. GCTA calculates the genetic similarity between subjects and uses the restricted maximum-likelihood approach to estimate narrow sense heritability (h^2^). An alternative approach based on phenotype correlation-genotype correlation (PCGC) regression has recently been developed to avoid potential bias introduced by GCTA when applied to case-control studies^14^. Here we apply both GCTA and PCGC methodologies to three glioma GWAS to enumerate the heritability accounted for by common genetic variation.

## Materials and Methods

### Ethics

Collection of blood samples and clinical information from subjects was undertaken with informed consent and relevant ethical review board approval in accordance with the tenets of the Declaration of Helsinki. Ethical committee approval for this study was obtained from relevant study centers [France: APHP ethical committee-CPP (comité de Protection des Personnes); Germany: Ethics Commission of the Medical Faculty of the University of Bonn and USA: University of Texas MD Anderson Cancer Institutional Review Board].

### Genome-wide association studies

We used three non-overlapping case-control series of Northern European ancestry which had been the subject of previous GWAS^8^,^9^; summarized in Supplementary Table 1. Briefly, the US GWAS was based on 1,247 cases (mean age 47 years) ascertained through the MD Anderson Cancer Center, Texas, between 1990 and 2008. Individuals with European ancestry from the Cancer Genetic Markers of Susceptibility (CGEMS) studies served as controls^8^,^15^,^16^. The French GWAS study comprised 1,423 patients with glioma ascertained through the Service de Neurologie Mazarin, Groupe Hospitalier Pitié-Salpêtrière Paris^9^. The controls were ascertained from the SU.VI.MAX (SUpplementation en VItamines et MinerauxAntioXydants) study of 12,735 healthy subjects (women aged 35–60 years; men aged 45–60 years)^17^. The German GWAS comprised 846 patients who underwent surgery for a glioma at the Department of Neurosurgery, University of Bonn Medical Center, between 1996 and 2008^9^. Control subjects were taken from three population studies: KORA (Co-operative Health Research in the Region of Augsburg; *n* = 371)^18^,^19^,^20^, POPGEN (Population Genetic Cohort; *n* = 595)^21^ and from the Heinz Nixdorf Recall study (*n* = 344)^22^. Genotyping quality control assessment was as previously described^8^,^9^ and all SNPs presented in this study passed the required thresholds. Duplicate samples were used to check genotyping quality. SNPs and samples with <95% SNPs genotyped were eliminated from the analyses. Genotype frequencies at each SNP were tested for deviation from the Hardy-Weinberg equilibrium (HWE) and rejected at *P* < 10^−4^.

### Statistical analysis

Consistent with our previous analysis^9^, quantile-quantile (Q-Q) plots for the German and US series showed some evidence of inflation (inflation factor *λ* = 1.15 and 1.11, respectively, based on the 90% least significant SNPs), however after correcting for population substructure using the top 10 principal-components in Eigenstrat, λ for all studies was ≤1.04 (Supplementary Figure 1). Therefore all heritability estimates were calculated adjusting for 10 principal components.

Artefactual differences in allele frequencies between cases and controls can contribute to the estimation of spurious genetic variation therefore for the current analysis we imposed a number of additional quality control measures to the dataset as advocated by Lee *et al.* when estimating heritability^23^. Using PLINK software^24^ we excluded SNPs in cases and controls that had a minor allele frequency (MAF) <0.01 or a HWE test with *P* < 0.05 (per-study exclusions = 60,932–62,065). Performing a differential missingness test between cases and controls we excluded SNPs with *P* < 0.05 (per-study exclusions = 25,002–60,235). In addition we excluded individuals having a relatedness score of >0.05. rs2736100 exhibited a HWE *P*-value of 0.01 in the German dataset but was still included in order to estimate the heritability attributable to known loci. This filtering resulted in the use of 263,905 SNPs common to the three case-control series (Supplementary Table 2). A total of 93 samples in the French series, 137 samples in the German series and 78 samples in the USA series were removed during quality-control steps for reasons including a failure to genotype, unknown duplicates, and closely related individuals, leaving 3,373 cases and 4,571 controls for heritability analysis.

Estimation of heritability was performed in GCTA using the methodology of Yang *et al.*^25^ and Lee *et al* 23. A genetic relationship matrix (GRM) of pairs of samples was used as input for the restricted maximum likelihood analysis to estimate the heritability explained by the selected set of SNPs. GCTA uses the disease prevalence to transform the estimated heritability to the liability scale. As previously advocated when calculating the heritability of a common lethal disease such as cancer^26^,^27^ we used the lifetime risk to transform the estimated heritability to the liability scale. Given glioma accounts for ~40% of all primary brain tumours^1^ and that GBM comprise 54.7% of gliomas^28^, and that the lifetime risk of brain and nervous system tumours is 0.62%^29^ we estimated the lifetime risk of all glioma, GBM and non-GBM tumors to be 0.24%, 0.13% and 0.11% respectively.

We estimated the heritability in GCTA under the following scenarios:

1. Heritability explained by the autosome. A single GRM is computed for all autosomal SNPs.
2. Heritability adjusting for incomplete LD between array SNPs and causal SNPs, following the procedure of Yang *et al*.^25^ and adjusting for a range of MAF thresholds of causal SNPs from <0.1 to <0.5.
3. Heritability explained by individual chromosomes. A GRM is computed for each chromosome individually and then fitting is done simultaneously for all chromosome GRMs using the REML approach.
4. Heritability explained by risk SNPs identified by GWAS as located within autosomal regions associated with glioma. For each risk SNP the heritability is estimated for all chromosomes simultaneously using the risk SNP genotype as a covariate. The heritability associated with the SNP is taken to be the difference between the heritability of the chromosome on which it is found as calculated with and without adjusting for the covariate.
5. Heritability explained by a) genic and intergenic, b) conserved (GERP > 2.0) and non-conserved (GERP < 2.0), c) functional (CADD > 10.0) and non-functional (CADD < 10.0) and d) transcription factor binding site occupying and non-occupying SNPs. For a-d separate GRMs are computed for SNPs in either of the two categories. Heritability was estimated simultaneously for the two GRM using the REML approach.

Estimation of genetic correlation between GBM and non-GBM glioma subtypes was carried out in GCTA using a bivariate REML analysis as per Lee *et al.*^30^, randomly assigning controls equally between the two glioma subtypes in each of the studies.

As advocated^31^ we estimated heritability using PCGC regression adjusting for population structure using a two-step procedure, where firstly the first 10 PCs (as computed in GCTA) are “cleaned” from the GRM, and are also subsequently included as fixed effects in the PCGC regression.

Meta-analysis under a fixed-effects model was conducted using standard methods. We calculated Cochran’s *Q* statistic to test for heterogeneity and the *I*^2^ statistic to quantify the proportion of the total variation that was caused by heterogeneity^32^.

GWAS array SNPs were functionally annotated using SeattleSeq Annotation 138^33^, making use of genomic evolutionary rate profiling (GERP)^34^ conservation metrics and combined annotation dependent depletion (CADD)^35^ scores. Conserved transcription factor bindings sites were predicted by the ‘tfbsCons’ track from the UCSC Genome Browser^36^, which searches for conserved elements in TransFac Matrix Database v4 after alignment in Human/Rat/Mouse.

## Results

Restricting our analysis to SNPs mapping to the autosomes and following quality control filtering a total of 263,905 SNPs common to the 3,373 cases and 4,571 controls from the three GWAS were analysed (Table 1).

### Variance explained by all autosomal SNPs

After transforming the data to account for lifetime risk and ascertainment the variance in liability of all glioma explained by the SNPs in each of the studies ranged from 0.23 to 0.35 (Table 1). Combining data from the three studies provides an estimate of the heritability of glioma of 0.25 (±0.03, Table 1). The confidence intervals of overall heritability estimates from the US, French and German series overlapped, arguing against population-specific differences affecting the estimates.

PCGC has recently been proposed to reliably estimate heritability from case-control GWAS data and to avoid bias introduced by GCTA when applied to case-control studies^31^. Heritability estimates for PCGC were 0.24 (±0.07, Supplementary Table 3). As this is very close to the estimate obtained from GCTA (0.25 ± 0.03), we restricted subsequent analysis to GCTA.

To adjust for the underestimate of heritability caused by the array SNPs not being in complete LD with the causal SNPs we followed the procedure of Yang *et al.*^25^. The MAF distribution of causal SNPs affects this estimate, and as we do not know the true distribution we calculate the adjustment for a range of MAF thresholds (Table 2). Assuming that causal SNPs and array SNPs have the same distribution (MAF threshold of 0.5) the adjusted heritability was calculated to be 31% (±3.8%) which is close to the unadjusted value of 25% (±3.0%). Conversely if causal SNPs are assumed to have MAF <0.1 then the adjusted heritability is 41% (±5.6%) which is significantly higher than the unadjusted value. While it is expected from neutral and selection theories of quantitative genetic variation that causal SNPs will on average have lower MAF than those on the array^37^ the exact distribution of MAF for causal SNPs in glioma is unknown.

Accumulating data have established that the glioma subtypes have different molecular profiles possibly resulting from different etiologic pathways, which might be shared by tumor subtypes or be type specific. To explore the possibility that common genetic variation *en masse* may impact differentially on the risk of glioma by subtype we estimated heritability for GBM and non-GBM glioma assuming that GBM accounts for ~55% of all glioma prevalence. Stratifying these data the variance in liability of GBM explained by the SNPs in each of the studies ranged from 0.21 to 0.48 with the pooled estimate being 0.26 (Table 1). The variance in liability of non-GBM explained by the SNPs in each of the studies was similar ranging from 0.22 to 0.29, with a pooled estimate of 0.25 (Table 1).

**Figure 1:**
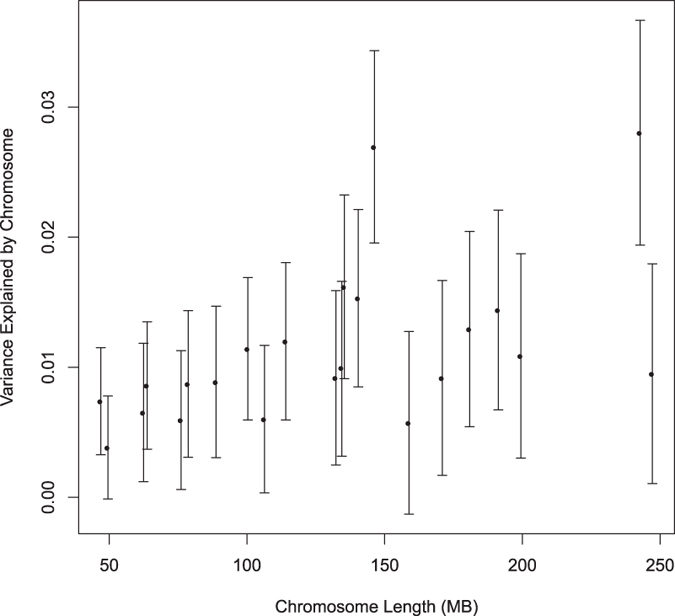
Estimate of the variance explained by each chromosome in the combined dataset as a function of chromosome size. The regression *R*^2^ was 0.29 (*P* = 0.010).

To gain insight into the underlying basis of the heritability associated with common variation we investigated the relative contribution of individual chromosomes (Supplementary Table 4). While for a trait such as height there is a strong linear relationship between chromosome length and the variance explained by the chromosome^13^, we observed only a modest relationship (R^2^ = 0.29 , *P* = 0.010, Fig. 1).

To determine the relative contribution of putatively functional vs non-functional SNPs to glioma heritability, we partitioned the variance explained by all the SNPs into different groupings, (Supplementary Table 5). There was little apparent difference in heritability estimates between SNPs mapping within genes versus those mapping outside of genes. Additionally, consistent with GWAS arrays being designed for tagging purposes many of the putative functional variants comprised a small fraction of the total and therefore heritability estimates inherently had a large error.

### Impact of known risk loci on variance

To determine the additive impact of the known GWAS risk loci on the heritability of glioma we estimated heritability by including the risk SNP as a covariate. The total estimated heritability of glioma explained by the seven risk loci is 1.6% (±2.6%), 1.3% (±3.9%) and 1.7% (±3.3%) for all glioma, GBM and non-GBM tumors respectively (Table 3). These estimates are substantially lower than the genetic variance associated captured by all the SNPs on the array. These data therefore suggest a large proportion of the heritability in glioma remains unaccounted for by currently identified risk SNPs.

## Discussion

Our results show for the first time that a substantial proportion (approximately 25%) of variation in the risk of developing glioma is associated with common SNPs (*i.e.* minor allele frequency, MAF >0.01) that are in LD with functional variants. These results are consistent with a highly polygenic model because we demonstrated variation across the entire genome. The methodology we have used here does not attempt to test the effects of single SNPs but tests their accumulated effects. It estimates the joint effect of genotyped SNPs and that effect reflects their LD with unknown functional variants, assuming that functional variants are in sufficient LD with the genotyped SNP for their contribution to heritability to be captured. Our estimate is based on realized relationships between very distant relationships thereby breaking up possible correlation (*i.e*. confounding) between genetic and environmental risk factors.

A strength of our study is that we were able to make use of data from three independent GWAS to derive combined heritability estimates, therefore minimising the possibility of biases in individual studies affecting study findings. Additionally we endeavoured to protect against differences in population structure between cases and controls affecting estimates by adjusting for 10 principal components in each of the three GWAS datasets. In the German dataset the GBM heritability estimate was significantly larger than in the other datasets hence we cannot entirely exclude the possibility of residual population structure differences affecting estimates.

Previous estimates of the heritability for glioma from segregation analysis, which have been based on analyses of pedigrees of 297 and 639 glioma probands have reported values of 0.68 and 0.66 for polygenic heritability respectively^38^,^39^. These estimates could differ from those that we report here for a number of reasons. Firstly, the narrow-sense heritability estimated in our analysis is simply the additive genetic variance as a proportion of the phenotypic variance. Therefore, it does not include non-additive epistatic gene-gene interactions, dominance effects or gene-environment interactions impacting on glioma risk.

Additionally, our estimate of heritability may provide a lower bound for narrow-sense heritability, due to imperfect LD between genotyped SNPs and unknown causal variants. Moreover, it has been argued that uneven linkage disequilibrium between genotyped SNPs and unknown causal variants could introduce bias in heritability estimates^40^. Furthermore, indels and structural variants were not considered, although some may be tagged. In addition, the proportion of variance explained by GWAS SNPs is underestimated by GCTA, since the model imposes a prior centered zero as the effect size of the SNPs used in calculation of the GRM. We have also assumed that inheritance is strictly Mendelian which excludes the possible contribution of *de novo* copy number variants or methylation status variants to glioma risk. It is therefore possible that our estimates of heritability are inherently conservative in terms of defining the contribution of the impact of inherited predisposition to glioma risk. Notwithstanding such caveats the magnitude of the estimated heritability in our study is such that this polygenic susceptibility contributes significantly to the development of glioma.

The liability threshold model upon which we have estimated heritability assumes the distribution of disease liability is unimodal. Provided there is no single unidentified genetic or a major environmental risk factor contributing to glioma this assumption will not be violated. Since no major disease gene for glioma has been identified and the only established environmental risk factor is ionizing radiation (accounting for very few cases), our estimates of heritability are unlikely to be significantly biased as a result of the assumed model of disease risk.

Not only do our findings provide quantification of the impact of common variation on glioma risk, they also provide a strong rationale for continuing to search for additional novel risk variants through GWAS-based strategies. Thus far, eight independent loci have been shown conclusively to be associated with glioma^8^,^9^,^10^,^41^. While the risk of glioma associated with these common variants is not insignificant (RRs of 1.2–1.4), collectively they underscore less than 5% of the entire genetic variance in risk of all forms of glioma. It is, therefore, likely that additional common low risk variants remain to be discovered and should be eminently harvestable in new larger GWAS or through further pooling of additional existing datasets. However, most novel risk variants yet to be discovered are likely to have more modest effect on glioma risk than those which have been so far discovered.

It is possible that some disease-causing variants which are very rare have a substantive effect on glioma risk but there is no reason to believe that much of the genetic variance is solely explained by a restricted number of high-risk mutations. Additional ongoing GWAS are therefore likely to be informative in refining estimates of heritability. Moreover, higher-density SNP genotyping would however provide a higher probability of LD with functional disease-causing variants thus potentially affording the capturing of a higher proportion of the genetic variance - provided the characteristics of disease-causing variants do not differ systematically from the genotyped SNPs (*e.g.* because of lower MAF).

Further advancements are likely to be made following the establishment of large consortia such as GLIOGENE^42^. Such initiatives not only provide the basis for GWAS with increased sample size, SNP coverage and number of SNPs taken forward to large-scale replication to aid in the identification of additional novel risk variants, but also facilitate pooling studies of existing GWAS data to importantly improve the standard error of the point estimate of the heritability of glioma.

Glioma is increasingly being viewed as a highly heterogeneous cancer. Primary and secondary forms of GBM are recognized, with secondary GBM developing through progression from low-grade diffuse astrocytomas or anaplastic astrocytomas. While usually indistinguishable histologically, distinct molecular pathways characterize the primary and secondary forms^43^. Notably, IDH1 mutations are commonly detectable in low-grade glioma and secondary GBM but are rare in primary GBMs^43^,^44^. Thus there are limitations on the interpretation of data obtained in our study in terms of the generalizability to all specific histological forms of glioma. Moreover, in recent years MRI scanning has revealed that low grade glioma can be incidentally detected in 0.2% of healthy individuals^43^,^44^ potentially impacting on the heritable risk. In this study we did however seek to address the impact of genetic variation on risk of glioma subtype by analysis of GBM and non-GBM glioma. A combined estimate of the genetic correlation between GBM and non-GBM in our datasets was 0.87, albeit with wide confidence limits (±0.22), suggesting that a significant complement of the heritable risk of glioma may be generic.

In summary, we report the first study to show that a large proportion of the heritability of developing glioma can be ascribed to common genetic variation. Moreover, it is the first to show biologically and unequivocally that the risk of glioma is highly polygenic. Our findings imply that very large sample sizes will be needed to detect novel loci with genome-wide significance and that the majority of additive genetic variation for glioma is not explained by rare variants that are not in LD with common SNPs.

## Additional Information

**How to cite this article**: Kinnersley, B. *et al.* Quantifying the heritability of glioma using genome-wide complex trait analysis. *Sci. Rep.* **5**, 17267; doi: 10.1038/srep17267 (2015).

## Supplementary Material

Supplementary Information

## Figures and Tables

**Table 1:** Estimated genetic variance of glioma explained by all SNPs.

Study	All glioma		GBM		Non-GBM	
	h^2^ (±S.E.)	*P*	h^2^ (±S.E.)	*P*	h^2^ (±S.E.)	*P*
France	0.23 (±0.05)	5.76 × 10^−6^	0.21 (±0.10)	0.017	0.22 (±0.06)	2.27 × 10^−5^
Germany	0.35 (±0.07)	2.18 × 10^−7^	0.48 (±0.09)	2.52 × 10^−7^	0.29 (±0.10)	0.0014
USA	0.23 (±0.04)	7.46 × 10^−9^	0.19 (±0.06)	8.87 × 10^−4^	0.26 (±0.06)	1.80 × 10^−5^
Combined	0.25 (±0.03)	1.15 × 10^−17^	0.26 (±0.05)	1.05 × 10^−8^	0.25 (±0.04)	1.24 × 10^−10^
I^2^/*P*_*het*_		25%/0.26		72%/0.03		0%/0.82
S.E., standard error.						

**Table 2:** Heritability of glioma adjusted for incomplete LD between causal SNPs and those used to compute the GRM.

MAF Threshold	h^2^ (±S.E.)	*P*
No adjustment	0.25 (±0.030)	1.13 × 10^−17^
0.5	0.31 (±0.038)	5.96 × 10^−16^
0.4	0.32 (±0.040)	1.25 × 10^−15^
0.3	0.33 (±0.042)	4.20 × 10^−15^
0.2	0.35 (±0.046)	2.11 × 10^−14^
0.1	0.41 (±0.056)	2.61 × 10^−13^

Various minor allele frequency (MAF) thresholds were used to simulate different possible MAF distributions of the causal SNPs.

**Table 3:** Estimates of the variance explained by individual glioma risk SNPs.

Locus	SNP	All glioma h^2^ (±S.E.)	GBM h^2^ (±S.E.)	Non-GBM h^2^ (±S.E.)
5p15.33	rs2736100	0.0012 (±0.011)	0.0053 (±0.016)	0.000017 (±0.013)
7p11.2	rs11979158	−0.00029 (±0.0099)	0.000093 (±0.015)	−0.00017 (±0.013)
7p11.2	rs2252586	0.00036 (±0.0099)	0.00096 (±0.015)	−0.00021 (±0.013)
8q24	rs4295627	0.0047 (±0.010)	0.00035 (±0.016)	0.0066 (±0.013)
9p21.3	rs4977756	0.0067 (±0.0095)	0.0036 (±0.015)	0.0025 (±0.012)
11q23.3	rs498872	0.0032 (±0.0095)	0.0025 (±0.013)	0.0082 (±0.012)
20q13.33	rs6010620	0.00034 (±0.0075)	0.00036 (±0.011)	−0.00021 (±0.0098)
Total		0.016 (±0.026)	0.013 (±0.039)	0.017 (±0.033)
S.E., standard error.				
