# Supplementary material for: Quantifying the heritability of glioma using genome-wide complex trait analysis
Source: Sci Rep. 2015 Dec 2;5:17267. doi: 10.1038/srep17267 (PMC4667278; doi:10.1038/srep17267)
Supplement: Supplementary Information [file srep17267-s1.pdf]

SUPPLEMENTARY INFORMATION

Quantifying the heritability of glioma using genome-wide complex trait analysis

Ben Kinnersely, Jonathan S Mitchell, Konstantinos Gousias, Johannes Schramm, Ahmed Idbaih, Marianne Labussière, Yannick Marie, Amythis Rahimian, H.-Erich Wichmann, Stefan Schreiber, Khe Hoang-Xuan, Jean-Yves Delattre, Markus M. Nöthen, Karima Mokhtari, Mark Lathrop, Melissa Bondy, Matthias Simon, Marc Sanson, Richard S Houlston

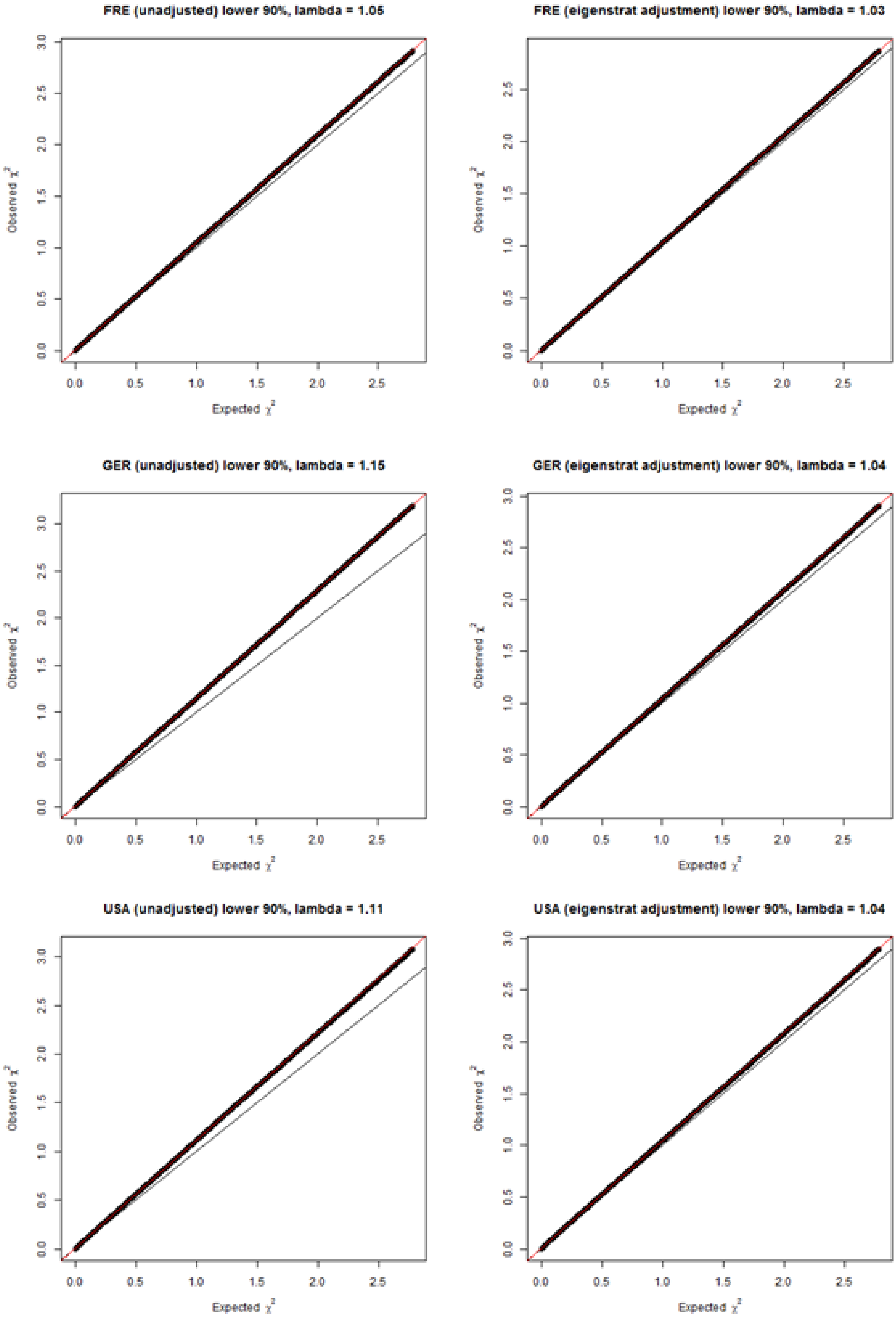

Supplementary Figure 1: Q-Q plot of test statistics (χ^2) for association with glioma. The black line represents the null hypothesis of no association. FRE, French GWAS; GER, German GWAS; USA, USA GWAS.

GWAS dataset	French	German	USA
**Cases (GBM/non-GBM)**	1,423 (430/993)	846 (431/415)	1,247 (652/595)
Study or Centre	Paris	Bonn	MD Anderson
Average age	48	51	47
Male/Female	816/607	474/372	771/476
Illumina Chip	HumanHap 660	HumanHap 660	HumanHap 610 Quad
**Controls**	1,190	1,310	2,236
Study	SU.VI.MAX	KORA, POPGEN & Heinz Nixdorf Recall	CGEMs
Illumina Chip	HumanHap 660	HumanHap 550	HumanHap 240+300; HumanHap 500

Supplementary Table 1: Summary characteristics of the three GWAS datasets. Sample numbers are post-QC (as described previously[1,2])

**GWAS dataset**	**French**	**German**	**USA**
Starting SNPs	425,190	425,190	425,190
**Exclusions**			
$P_{HWE} < 0.05$	62,065	63,429	60,932
$P_{missing} < 0.05$	25,002	60,235	43,364
**Consistent final set**	263,905	263,905	263,905

Supplementary Table 2: SNP quality control for GCTA. HWE, Hardy Weinberg equilibrium. $P_{missing}$ is a per-SNP test for significant differences in missing data between cases and controls.

	All glioma	
**Study**	**h^2 (± S.E.)**	***P***
**France**	0.14 (± 0.08)	
**Germany**	0.36 (± 0.10)	
**USA**	0.25 (± 0.06)	
**Combined**	0.24 (± 0.04)	$6.42x10^{-8}$
**I^2/ P_{het}**		37.5%/0.20

Supplementary Table 3: PCGC heritability estimates. S.E., standard errors.

Chromosome	Size (bp)	h^2 (± S.E.)
1	247,199,719	0.0095 (± 0.0085)
2	242,751,149	0.028 (± 0.0086)
3	199,446,827	0.011 (± 0.0079)
4	191,263,063	0.014 (± 0.0077)
5	180,837,866	0.013 (± 0.0075)
6	170,896,993	0.0092 (± 0.0075)
7	158,821,424	0.0057 (± 0.0070)
8	146,274,826	0.027 (± 0.0074)
9	140,442,298	0.015 (± 0.0068)
10	135,374,737	0.016 (± 0.0071)
11	134,452,384	0.010 (± 0.0067)
12	132,289,534	0.0092 (± 0.0067)
13	114,127,980	0.012 (± 0.0060)
14	106,360,585	0.0060 (± 0.0057)
15	100,338,915	0.011 (± 0.055)
16	88,822,254	0.0089 (± 0.0058)
17	78,654,742	0.0087 (± 0.0056)
18	76,117,153	0.0059 (± 0.0053)
19	63,806,651	0.0086 (± 0.0049)
20	62,435,965	0.0065 (± 0.0053)
21	46,944,323	0.0074 (± 0.0041)
22	49,528,953	0.0038 (± 0.0040)
Total		0.25 (± 0.031)

Supplementary Table 4: Estimates of the variance explained by individual chromosomes.
S.E., standard error.

Group	Category	N (SNPs)	h^2 (± S.E.)
1	Within gene	124,824	0.13 (± 0.023)
	Outside gene	138,814	0.12 (± 0.024)
2	Conserved (GERP > 2)	27,982	0.041 (± 0.020)
	Not conserved (GERP < 2)	232,727	0.21 (± 0.034)
3	CADD score > 10	26,511	0.015 (± 0.020)
	CADD score < 10	237,126	0.24 (± 0.033)
4	Tfbs occupied	5,815	0.016 (± 0.010)
	Tfbs unoccupied	257,823	0.24 (± 0.031)

Supplementary Table 5: Heritability estimates for different functional characteristics of SNPs. CADD, combined annotated dependent depletion; GERP, genomic evolutionary rate profiling; Tfbs, transcription factor binding site. S.E., standard error. The enrichment statistic details the relative contribution to heritability within the group after adjusting for SNP number.
